# Structural details of monoclonal antibody m971 recognition of the membrane-proximal domain of CD22

**DOI:** 10.1016/j.jbc.2021.100966

**Published:** 2021-07-14

**Authors:** June Ereño-Orbea, Xianglei Liu, Taylor Sicard, Iga Kucharska, Wei Li, Dorota Borovsky, Hong Cui, Yang Feng, Dimiter S. Dimitrov, Jean-Philippe Julien

**Affiliations:** 1 Program in Molecular Medicine, The Hospital for Sick Children Research Institute, Toronto, Ontario, Canada; 2 Ikerbasque, Basque Foundation for Science, Bilbao, Bizkaia, Spain; 3 Division of Infectious Diseases, Department of Medicine, University of Pittsburgh, Pennsylvania, USA; 4 Department of Biochemistry, University of Toronto, Toronto, Ontario, Canada; 5 Department of Immunology, University of Toronto, Toronto, Ontario, Canada; 6 Protein Interactions Group, Center for Cancer Research Nanobiology Program, Center for Cancer Research, National Institutes of Health, Frederick, Maryland, USA

**Keywords:** antibody, B cell, CD22, m971, CAR-T cell, crystal structure, B-ALL, B-cell acute lymphoblastic leukemia, BLI, biolayer interferometry, CARs, chimeric antigen receptors, CAR-T cell, chimeric antigen receptor T cell, CD22, cluster of differentiation-22, ECD, extracellular domain, Fab, fragment antigen-binding, GAG, glycosaminoglycan, HCDRs, heavy-chain complementarity-determining regions, HEK 293S, HEK 293 GnT I^−/−^, Ig, immunoglobulin, ITIMs, intracellular tyrosine-based inhibitory motifs, MSLN, mesothelin, Siglec, sialic acid–binding immunoglobulin-like lectin, SLS, static light scattering, T_m_, melting temperature, T_onset_, onset aggregation temperature

## Abstract

Cluster of differentiation-22 (CD22) belongs to the sialic acid–binding immunoglobulin (Ig)-like lectin family of receptors that is expressed on the surface of B cells. It has been classified as an inhibitory coreceptor for the B-cell receptor because of its function in establishing a baseline level of B-cell inhibition. The restricted expression of CD22 on B cells and its inhibitory function make it an attractive target for B-cell depletion in cases of B-cell malignancies. Genetically modified T cells with chimeric antigen receptors (CARs) derived from the m971 antibody have shown promise when used as an immunotherapeutic agent against B-cell acute lymphoblastic leukemia. A key aspect of the efficacy of this CAR-T was its ability to target a membrane-proximal epitope on the CD22 extracellular domain; however, the molecular details of m971 recognition of CD22 have thus far remained elusive. Here, we report the crystal structure of the m971 fragment antigen-binding in complex with the two most membrane-proximal Ig-like domains of CD22 (CD22_d6–d7_). The m971 epitope on CD22 resides at the most proximal Ig domain (d7) to the membrane, and the antibody paratope contains electrostatic surfaces compatible with interactions with phospholipid head groups. Together, our data identify molecular details underlying the successful transformation of an antibody epitope on CD22 into an effective CAR immunotherapeutic target.

A rapidly expanding immunotherapy to treat hematological cancer uses *ex vivo*–modified mature T lymphocytes that are engineered to express chimeric antigen receptors (CARs) specific for a targeted antigen on cancer cells (1). The engineered CAR is capable of redirecting T cells (chimeric antigen receptor T cell [CAR-T cells]) to specifically target and destroy malignant cells expressing the antigen without major histocompatibility complex restriction (2, 3). In the case of B-cell malignancies, such as B cell–associated leukemias and lymphomas, CD19 is a compelling target for CAR-T cell–based therapies because of its restricted expression to the B-cell lineage, thus reducing off-target global cytotoxic effects. To date, there are two anti-CD19 CAR-T cells approved for use by the US Food and Drug Administration for the treatment of pediatric acute lymphoblastic leukemia and adult diffuse large B-cell lymphoma (4). Although complete tumor regression (70–90% for B-cell acute lymphoblastic leukemia [B-ALL]) can be achieved in a substantial fraction of patients, anti-CD19 CAR-T cell therapy has also suffered resistance in some cases because of the loss of expression of the antigen (5, 6, 7, 8).

Cluster of differentiation-22 (CD22) represents an alternative target antigen for CAR-T cells in B-cell malignancies. Indeed, CAR-T cells targeting CD22 have shown potent antineoplastic effects in a phase 1 clinical trial enrolling patients who failed to achieve remission in the CD19 CAR-T cell therapy protocol (9, 10). CD22 is a transmembrane glycoprotein expressed on B cells that commonly retain expression in CD19_neg_ tumors (9, 10). The canonical function of CD22 is to dampen the activating signal of the B-cell receptor. As part of the sialic acid–binding immunoglobulin (Ig)-like lectin (Siglec) family, the extracellular domain (ECD) of CD22 recognizes glycans terminated in α-2,6-sialic acid, and its binding site is located at the most membrane-distal domain (d1) (11). The CD22 ECD is composed of seven Ig-like domains (d1–d7) and contains 12 predicted N-linked glycans (Fig. 1). Binding to sialic acid results in phosphorylation of the CD22 intracellular tyrosine-based inhibitory motifs, subsequent recruitment of tyrosine-protein phosphatase SHP-1, and the dampening of the B-cell response (12). CD22 itself is covered with N-linked glycans terminated in α-2,6-sialic acid and forms homo-oligomers on the surface of B cells (13). The tilted conformation adopted by CD22 and the location of the binding site on d1 has been proposed to favor *cis*-interactions and the formation of these nanoclusters (11).

**Figure 1:**
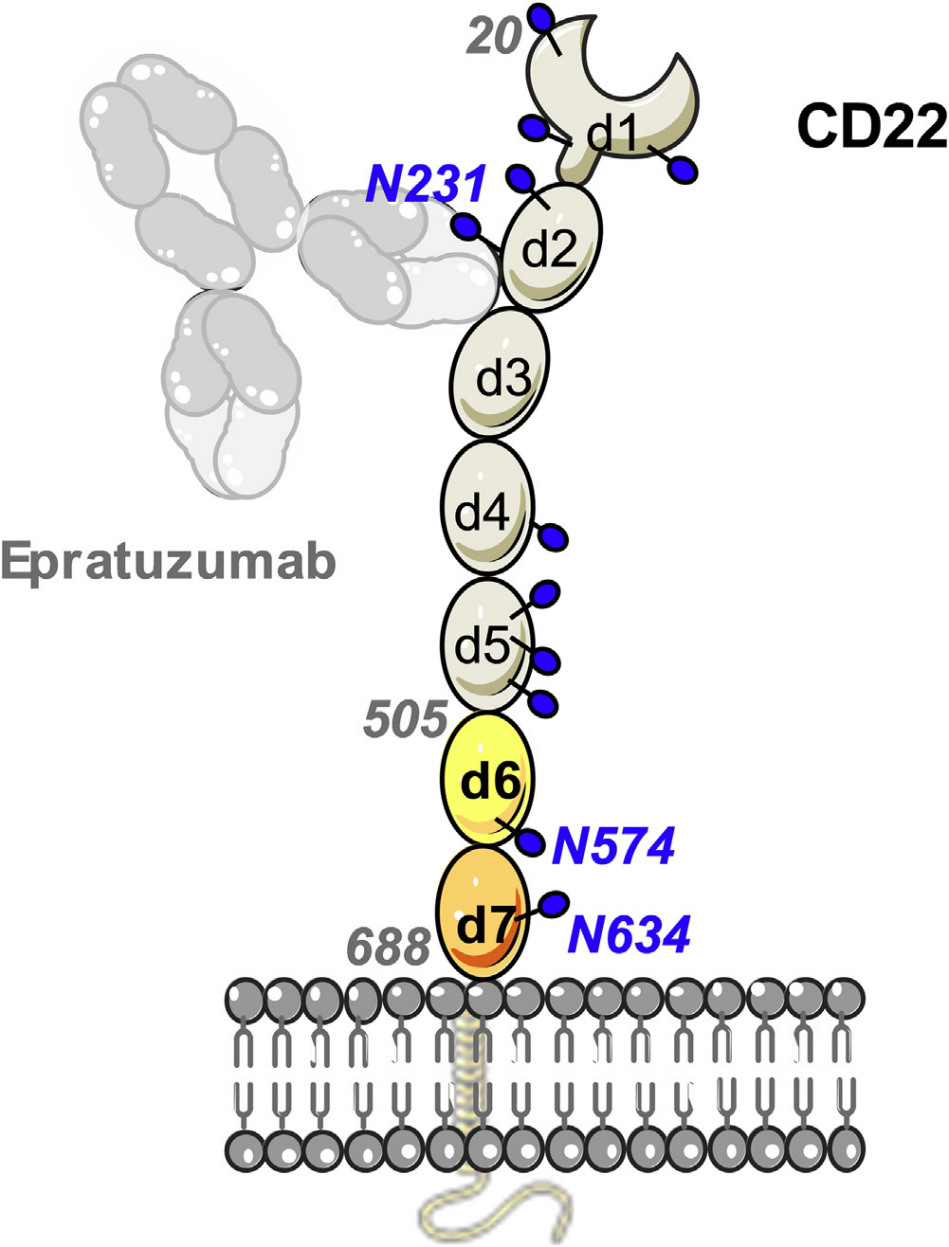
**Schematic representation of CD22 on the cell surface bound by therapeutic antibody epratuzumab.** The extracellular domain (ECD) is comprised of seven Ig domains (d1–d7). CD22 is a membrane glycoprotein with 12 N-linked glycans (*blue spheres*) on the ECD. The CD22_d6–d7_ construct (residues 505–688) contains domains d6 (in *yellow*) and d7 (in *wheat*), which are predicted to have two N-linked glycans on residues N574 and N634. Epratuzumab binds the CD22 d2/d3 interface close to N-linked glycan N231 (11). Ig, immunoglobulin.

The essential components of a CAR include the extracellular antigen-binding domain, usually a single-chain fragment variable, a transmembrane, and hinge region that anchors the receptor on the cell surface and projects the single-chain fragment variable out to the ECD, and intracellular signaling motifs from the T-cell receptor (such as CD3ξ, CD28, 4-1BB, or OX40) that are triggered upon antigen engagement. Thus, CARs are designed to transduce antigen-recognition events into signaling cascades that evokes T-cell effector functions, such as the secretion of cytotoxic factors and proinflammatory cytokines. Previous studies on the development of anti-CD22 CAR-T cells showed the relevance of targeting a membrane-proximal epitope on CD22 ECD (10). Interestingly, almost all CD22-directed monoclonal antibodies (mAbs) reported to date recognize membrane-distal epitopes on CD22; for example, the epitope of epratuzumab was structurally delineated at the interface of the CD22 d2 and d3 domains (Fig. 1) (11). On the other hand, mAb m971 was previously shown to target a membrane-proximal epitope on CD22, and a CAR-T cell using the m971 binding specificity achieved superior antileukemic effects in preclinical models of B-ALL in comparison with anti-CD22 CAR-T cells of similar affinity targeting membrane-distal regions (10).

To provide molecular insights into antibody recognition that leads into the development of effective anti-CD22 CAR-T cells, we structurally and biophysically delineated the binding site of m971 on CD22 (14). Our crystal structure delineates the three-dimensional architecture of the most membrane-proximal Ig domains d6 and d7 of CD22 (CD22_d6–d7_) and reveals that m971 binds at the membrane-most base of CD22 to mediate its antileukemic effects. Together, our data contribute molecular principles of antigen recognition underlying the development of potent CAR-T cells.

## Results

### Structure of CD22 d6–d7 domains

To delineate the m971 epitope at high resolution, we obtained crystals of the membrane-proximal domains d6 and d7 of the extracellular portion (ECD) of human CD22 (CD22_d6–d7_) in complex with the m971 fragment antigen-binding (Fab), which diffracted to 2.4 Å resolution (Table 1). The antigen–antibody structure was solved by molecular replacement using the m971 Fab as an initial search model, which was derived from its 1.6 Å resolution crystal structure in complex with the crystallization chaperone anti-kappa V_H_H domain (15) (Table 1). The phases obtained were of sufficient quality for manual building of the d6 and d7 domains of CD22. CD22 d6 showed overall weaker electron density compared to d7, presumably because of lower crystal packing interactions and thus more flexibility in the crystal lattice.

**Table 1:** Crystallographic data collection and refinement statistics

Attributes	m971 Fab–V_H_H complex	CD22_d6–d7_–m971 Fab complex
PDB ID	7O4Y	7O52
Data collection statistics		
Wavelength (Å)	0.97951	1.03320
Resolution range (Å)	39.13–1.60 (1.66–1.60)	48.87–2.41 (2.49–2.41)
Space group	P3_1_	I222
Unit cell a, b, c (Å)	73.9, 73.9, 98.8	61.5, 119.7, 241.7
α, β, γ (°)	90, 90, 120	90, 90, 90
Total reflections	329,897	461,724
Unique reflections	79,795 (12,852)	35,031(5468)
Multiplicity	4.1 (3.9)	13.1 (12.4)
Completeness (%)	99.8 (99.4)	99.5 (95.2)
Mean I/σI	17.3 (2.3)	20.2 (1.5)
Wilson B-factor (Å^2^)	20.9	60.4
R_merge_	0.043 (0.436)	0.087 (0.825)
R_pim_	0.024 (0.249)	0.025 (0.236)
CC_1/2_	99.9 (77.7)	99.9 (58.4)
Refinement statistics		
Resolution (Å)	39.13–1.60 (1.66–1.60)	48.87–2.41 (2.49–2.41)
R_work_	0.176 (0.253)	0.226 (0.349)
R_free_	0.208 (0.266)	0.265 (0.407)
Number of nonhydrogen atoms	4932	4746
Macromolecules	4227	4652
Heteroatom	-	57
Solvent	705	37
RMS (bonds)	0.011	0.002
RMS (angles)	1.42	0.60
Ramachandran statistics:		
Favored (%)	97.9	95.9
Allowed (%)	2.0	3.5
Outliers (%)	0	0.5
Rotamer outliers (%)	0.6	0.0
Average B (Å^2^)		
Macromolecules	25.6	69.8
Heteroatoms	-	93.5
Solvent	34.6	55.7
Clashscore	2.3	3.8

**Figure 2:**
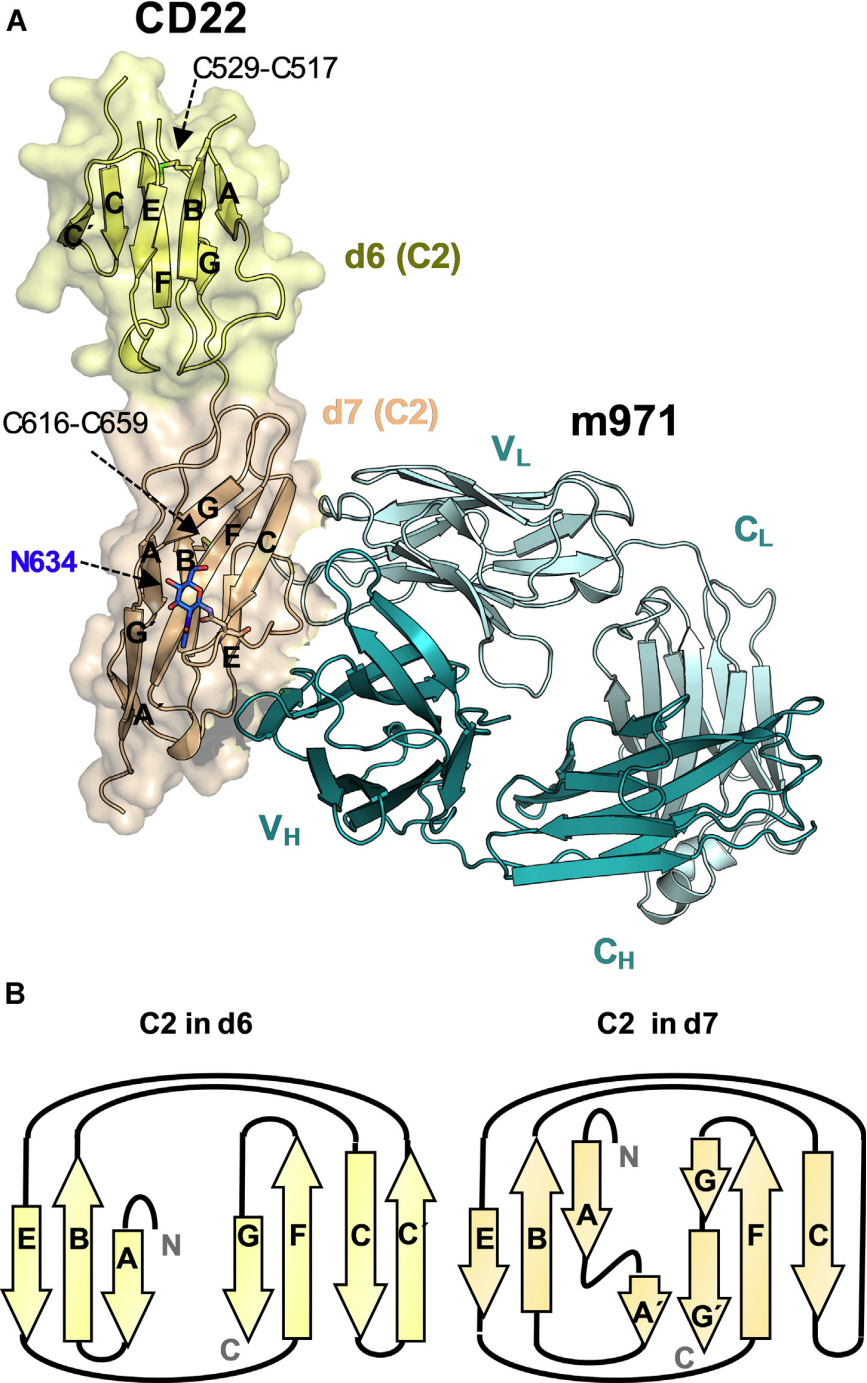
**Three-dimensional structure of d6 and d7 Ig-like domains of CD22 in complex with m971 Fab.***A*, crystal structure of CD22_d6–d7_ in complex with m971 Fab. D6 (in *yellow*) and d7 (in *wheat*) adopt a C2-type Ig-domain fold. The N-acetylglucosamine at N634 in d7 is shown as *blue sticks*. The internal disulfide linkage in each Ig domain (between C529 and C517 in d6; between C616 and C659 in d7) is represented as *sticks*. Residues 574 to 580 in d6 were not modeled because of high flexibility of this loop. m971 recognizes an epitope on d7 comprising of β-strand C, and loops C–E and E–F. The heavy chain (*dark cyan*) and light chain (*cyan*) of m971 are represented as *cartoon*s. *B*, topology diagram of d6 and d7 C2-type Ig-like domains. The β-sheets are formed by strands ABE and C'CFG in d6 and by strands A'ABE and CFGG' in d7. Fab, fragment antigen-binding; Ig, immunoglobulin.

As predicted from the primary sequence, the two consecutive CD22 d6 and d7 Ig domains possess a C2-type Ig fold, each with one intradomain disulfide linkage (C529-C517 in d6 and C616-C659 in d7) (Fig. 2). In contrast, the most membrane-proximal domain of the related Siglec-4 (MAG) showed a C1-type fold and two intradomain disulfide linkages (Fig. S1) (16). Thus, the CD22_d6–d7_ structure highlights differences in the structures adopted by membrane-proximal Ig domains among the Siglec family. In the cocrystal structure, CD22 residues 574 to 580 located in d6 could not be built because of apparent high flexibility in this loop. Only one of the two predicted N-linked glycans in CD22_d6–d7_ was visible in the electron density and was built at N634 in d7, with one residual N-acetylglucosamine moiety left after endoglycosidase H deglycosylation of the recombinant CD22_d6–d7_ expressed in HEK 293 GnT I^−/−^ (HEK 293S) cells before crystallization (Fig. 2).

### m971 Fab recognizes the CD22 d7 domain

**Figure 3:**
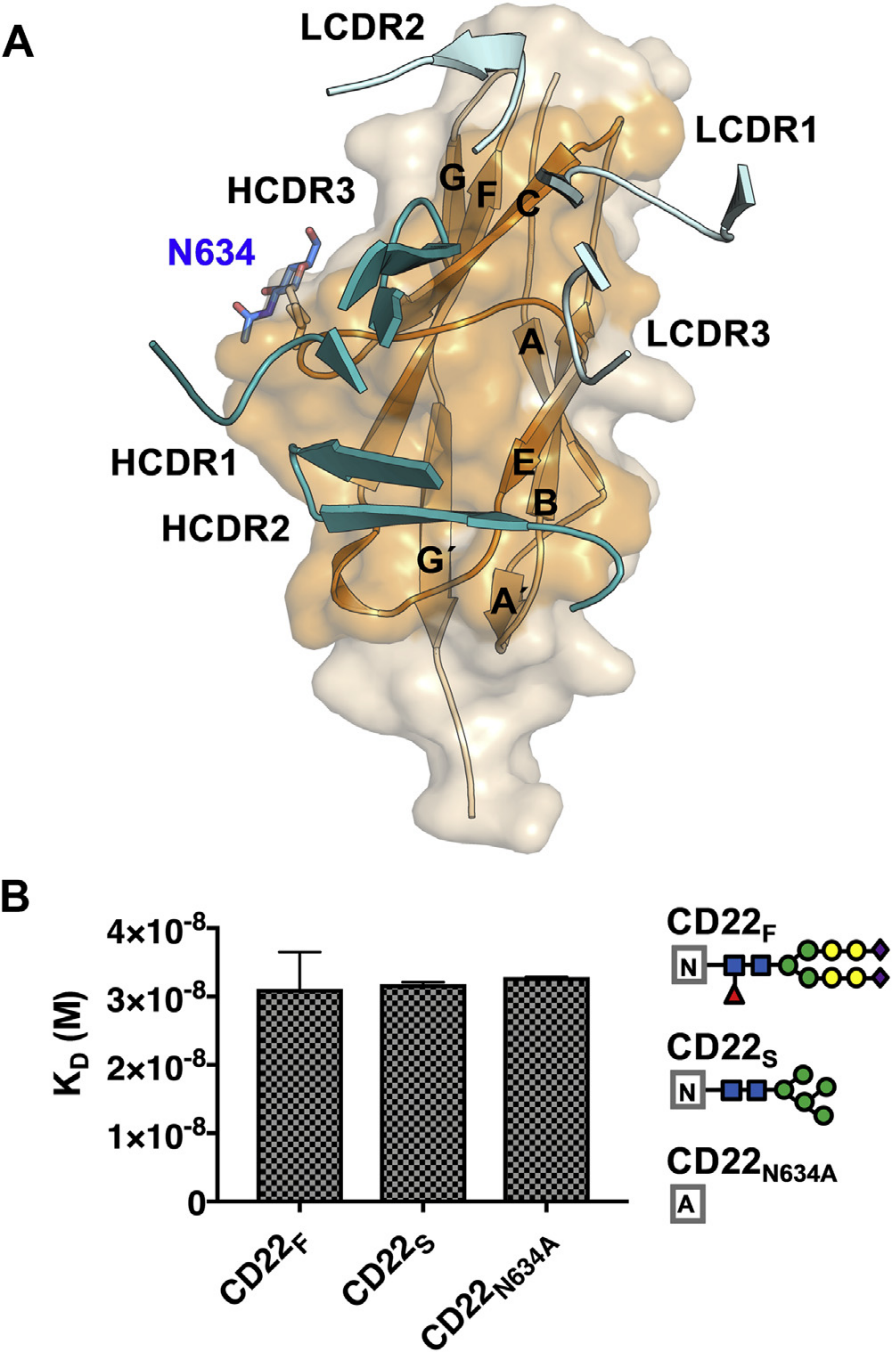
**m971 epitope delineation on CD22.***A*, CDR loops from m971 interacting with the CD22 d7 domain (*wheat*). CDR loops from the variable heavy chains (*deep teal*) HCDR1, HCDR2, and HCDR3 interact with β-strand C. LCDR1 and LCDR2 (*cyan*) interact with loops C–E and E–F of d7. *B*, binding affinity of m971 Fab to CD22 ECD bearing complex N-linked glycans from expression in HEK 293F cells (CD22_F_), more homogeneous and high-mannose glycans from expression in HEK 293S cells (CD22_S_), and CD22 ECD containing the N634A mutation to remove this N-linked glycosylation site (CD22_N634A_). K_D_’s are indicated with the SEM and derive from two independent measurements. Each symbol represents a specific monosaccharide: N-acetylglucosamine (*blue rectangle*), fucose (*red triangle*), mannose (*green circle*), galactose (*yellow circle*), and Neu5Ac (*purple diamond*). CDR, complementarity-determining region; ECD, extracellular domain; HCDR, heavy-chain complementarity-determining region; LCDR, light-chain complementarity-determining regions.

The cocrystal structure revealed that the m971 Fab binds to the base of the CD22 d7 domain (Fig. 2). Comparison of the variable domain of the CD22-liganded and -unliganded crystal structures of m971 Fab indicated that its paratope is largely preconfigured for binding its epitope (RMSD of 0.33 Å) (Fig. S2). The three heavy-chain complementarity-determining regions (HCDRs 1, 2, and 3) and the light-chain complementarity-determining regions 1 and 2 of m971 interact with the C β-strand, and loops C–E and E–F of CD22 d7 (Fig. 3). The buried surface area on the antigen is extensive (990 Å^2^) and is primarily mediated by the heavy chain (722 Å^2^) and less by the light chain (268 Å^2^) (Table S1). As opposed to mAb epratuzumab, in which the epitope includes an N-linked glycan at position N231 on d2 (Fig. 1), m971 does not include the N-linked glycan on d7 (N634). This result was further corroborated by binding kinetics experiments with a KO of the N634 glycosylation site at d7 (N634A mutation), which resulted in a binding affinity similar to WT CD22 (Fig. 3). Together, our data indicate that m971 recognizes the closest ECD to the membrane of CD22 and that N-linked glycosylation on CD22 does not impact the ability to access its epitope.

### A Y^H52A^R mutation on m971 Fab increases affinity toward CD22 ECD

**Figure 4:**
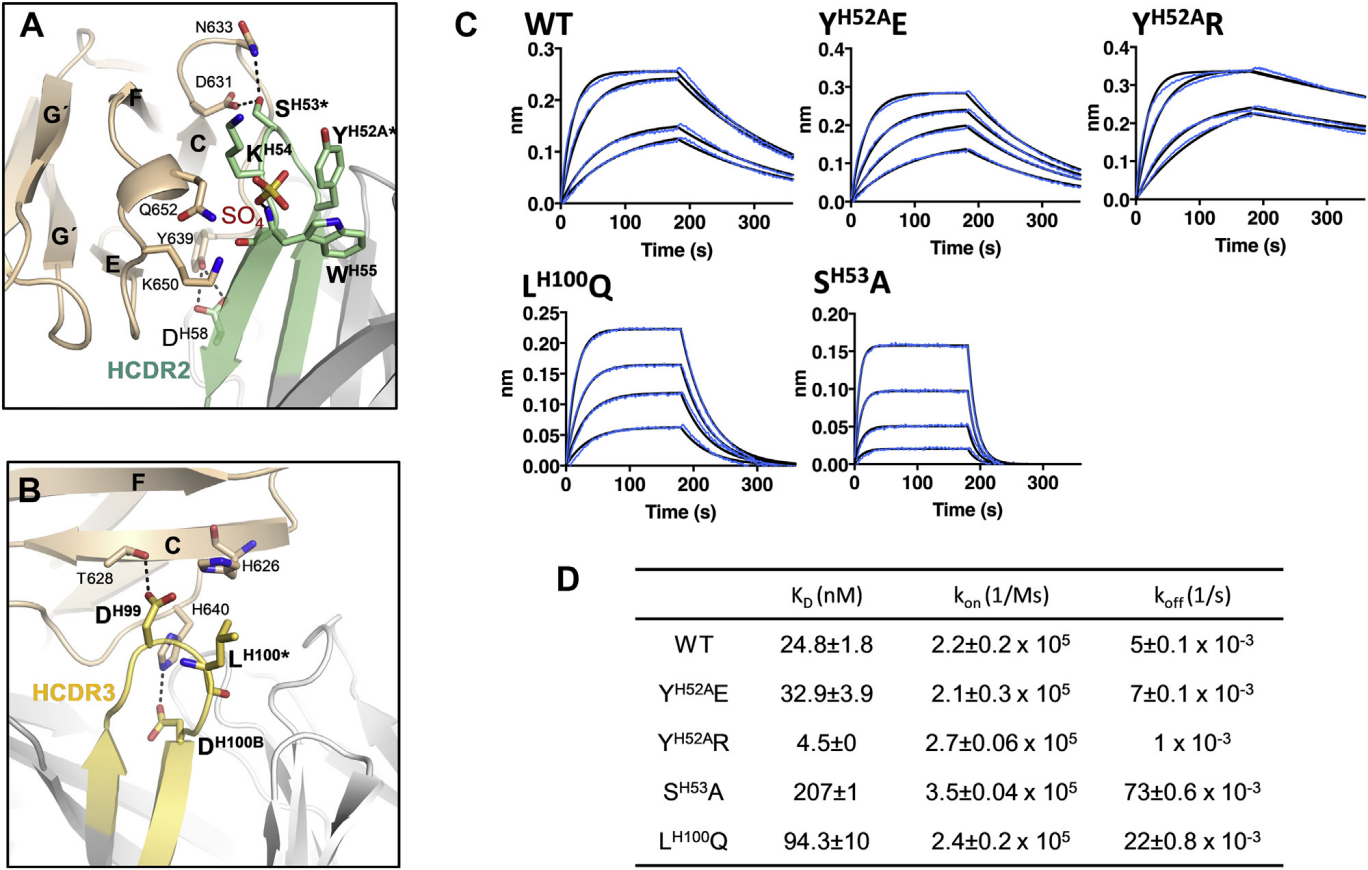
**Mutations in the m971 paratope impact binding affinity to CD22.***A*, interactions of the m971 HCDR2 with the CD22 d7 domain (*wheat*). Sulfate ion site III (*red* and *yellow sticks*) forms an H-bond with the amide backbone of W^H55^. *B*, interactions of the m971 HCDR3 with the CD22 d7 domain (*wheat*). *Dashed black lines* in panels *A* and *B* represent polar contacts. Mutated residues in HCDR2 and HCDR3 are highlighted with an *asterisk*. *C*, biolayer interferometry (BLI) data showing binding of WT and mutant m971 Fabs (200, 100, 50, and 25 nM concentrations) to CD22_d1–d7_. Raw experimental data are in *blue*, and fitted curves for binding kinetics determination are in *black*. *D*, K_D_, k_on_, and k_off_ values describing the binding of WT and mutant m971 Fabs to CD22_d1–d7_. K_D_, k_on_, and k_off_ mean values are presented with their SEM derived from two independent BLI measurements. Fab, fragment antigen-binding; HCDR, heavy-chain complementarity-determining region.

**Figure 5:**
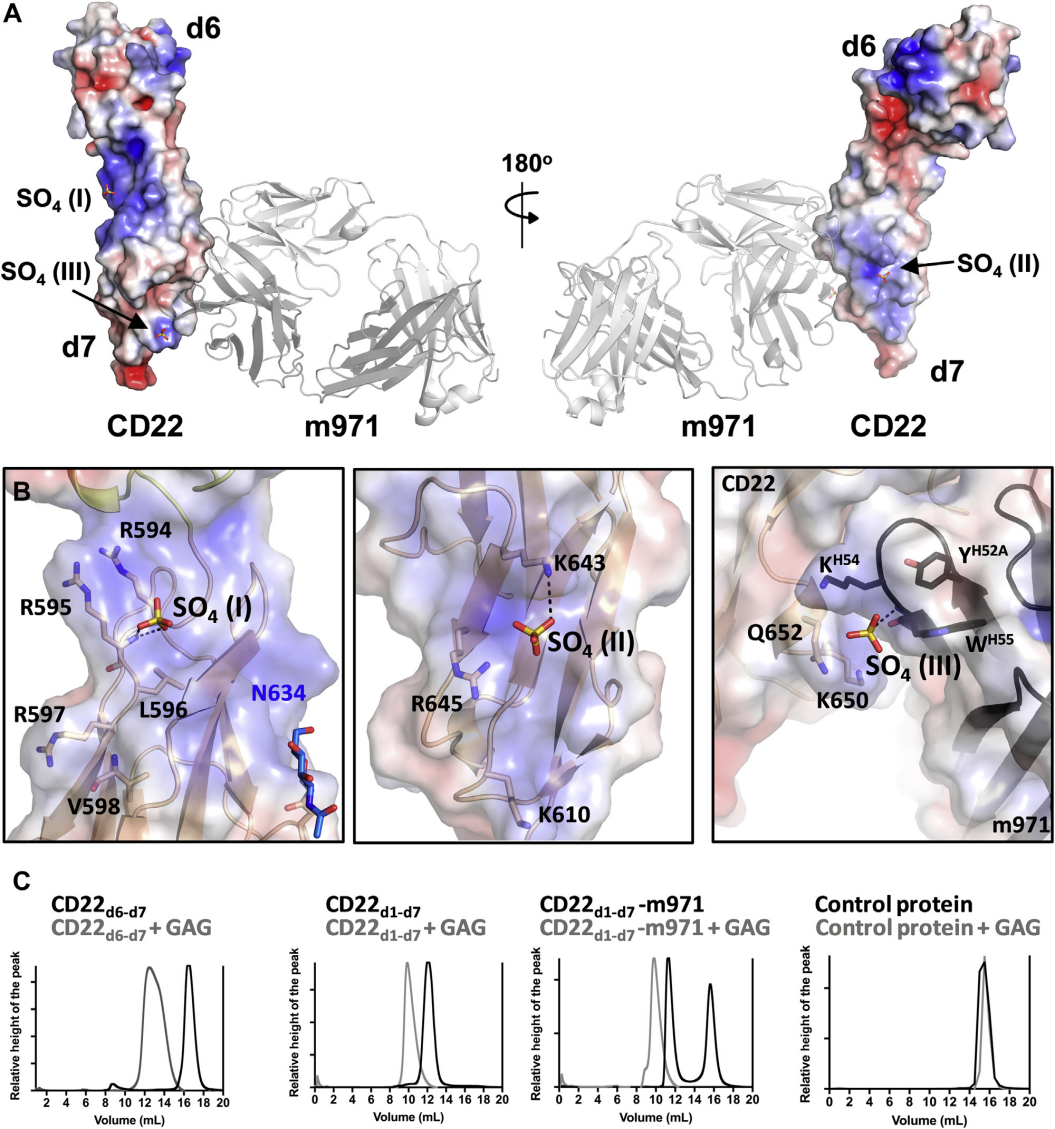
**Location of sulfate ions I, II, and III in the crystal structure of CD22**_**d6–d7**_**.***A*, electrostatic surface representation of CD22_d6–d7_ showing the localization of sulfate ions (SO_4_ I, II, and III; shown as *sticks*) around the CD22 d7 domain in the crystal structure. m971 Fab bound to CD22 is represented in a *cartoon* (*gray*). *B*, main interactions between SO_4_ I (*left*), SO_4_ II (*middle*), and SO_4_ III (*right*) with CD22. SO_4_ III (*right*) also interacts with the HCDR2 of m971 Fab. The calculation of the surface electrostatics was made with the APBS software (39) and prepared using PyMOL (40) and are displayed on a scale of −5 kT/e (*red*) to 5 kT/e (*blue*). The electrostatic surface is represented with transparency to visualize the *cartoon* and *sticks* of CD22 residues underneath. *C*, chromatograms from Superdex 200 Increase 10/300 column, after running CD22_d6–d7_, CD22_d1–d7_, CD22_d1–d7_-m971 Fab, or the epratuzumab Fab control protein alone (*black*) or with 10× molar excess of GAG (*gray*). The protein eluting at 16 ml in the chromatogram of CD22_d1d7_–m971 (*black line*) corresponds to a molar excess of m971 Fab. Fab, fragment antigen-binding; GAG, glycosaminoglycan; HCDR, heavy-chain complementarity-determining region.

To probe the CD22–m971 binding interface, we next introduced single-point mutations by site-directed mutagenesis in the m971 Fab. Residues S^H53^ in HCDR2 and L^H100^ in HCDR3 were mutated in attempts to alter the affinity toward CD22 (Fig. 4). Interestingly, the m971 paratope contains a basic patch in HCDR2 (Fig. S2). Based on this observation, we decided to mutate Y^H52A^ to a basic (R) or acidic (E) residue in HCDR2 to determine the effect of gaining positive or negative charge at the interface between m971 and CD22 (Fig. 5). Measuring intrinsic fluorescence simultaneously with static light scattering (SLS) through a temperature ramp, we found that all mutants exhibited similar onset melting temperatures (T_m_) and onset aggregation temperatures (T_onset_), in comparison with WT m971, under three different buffer conditions (pH 5.6, 7.4, and 9) (Fig. S3).

In biolayer interferometry (BLI) experiments, the affinity of WT m971 to the full-length ECD of CD22 (CD22_d1–d7_) was calculated to be in the low nanomolar range (25 nM; Fig. 4), which is comparable with the affinity previously measured for the IgG by flow cytometry (75 nM) (14). All m971 Fab mutants tested showed measurable binding to CD22_d1–d7_. As expected from our structural studies, the S^H53^A mutation in HCDR2 decreased the binding affinity of m971 Fab to CD22 by approximately 8-fold (K_D_ = 207 nM), primarily because of a faster off-rate (Fig. 4). Indeed, hydrogen bonds formed between m971 S^H53^ and CD22 D631 and N633 would be lost with an alanine mutation (Fig. 4). Similarly, substitution of the hydrophobic L for Q, a polar residue, at position 100 in the HCDR3 (L^H100^Q mutation) resulted in an ∼4-fold decrease in binding affinity (K_D_ = 94 nM) to CD22_d1–d7_ (Fig. 4). Interestingly, while substitution of the aromatic Y^H52A^ residue for the negatively charged E residue in HCDR2 (Y^H52A^E mutation) minimally affected its binding affinity (K_D_ = 33 nM), substitution to positively charged R (Y^H52A^R) increased the binding affinity ∼5-fold to CD22_d1–d7_ (K_D_ = 5 nM). The observed increase in the affinity for Y^H52A^R mutant results from a slower off-rate (Fig. 4). We further analyzed the activity of a CAR-T cell containing the variant Y^H52A^R (Fig. S4). However, the slight gain in affinity for CD22 did not result in increased cell killing. Together, we conclude that our mutagenesis data of the m971 paratope validates the CD22_d6–d7_-m971 Fab cocrystal structure.

### Anion-binding sites on the surface of the d7 domain of CD22

Three strong spherical electron densities close to positively charged surfaces on the CD22 d7 domain were observed in the electron density map (Fig. S5). Based on their shape, size, environment, and high concentration of ammonium sulfate in the crystallization buffer, we interpreted these densities as sulfate ions (SO_4_ I, II, and III) (Fig. 5). The B-factors of the sulfate ions range from 87 to 108 Å^2^, only slightly higher than the overall B-factor of the refined structure (69 Å^2^).

Interestingly, SO_4_ III is located at the interface between the basic patches of the CD22 d7 domain and the m971 HCDR2 and is stabilized by one hydrogen bond with the amide backbone of m971 W^H55^ (Fig. 5 and Fig. S5). On the other hand, SO_4_ II is located near a basic patch formed by β-strands B and E of the CD22 d7 domain, with H-bond interactions with T613, K643, and R645 (Fig. 5). SO_4_ I is located at the N-terminal region of the CD22 d7 domain, next to a basic motif (593-PRRLRV-598) and interacts with the amide backbone of R597 (Fig. 5 and Fig. S5). Interestingly, this motif resembles a consensus motif XBBXBX (B being the basic amino acids arginine, lysine, or histidine and X being one of a range of aliphatic/aromatic amino acids) known to bind negatively charged heparin sulfate (17). The presence of putative heparin sulfate-binding motifs in CD22 appears conserved across mammals (Fig. S6) and suggests that heparin sulfate may play a role in CD22 signal transduction and/or in protein–protein interactions. To probe the ability of the CD22 membrane-proximal region to bind negatively charged polysaccharides, we tested binding of CD22_d6–d7_ to glycosaminoglycan (GAG) of 14 to 16 kDa molecular weight. For this experiment, we mixed CD22 (CD22_d6–d7_ or CD22_d1–d7_ constructs) in the presence or absence of m971 Fab, with 10× molar excess of GAG and tested the complex formation by size-exclusion chromatography (Fig. 5). The shift of the elution peak to an earlier volume suggested complex formation between CD22 and GAG, consistent with the presence of a putative heparin sulfate-binding site. Moreover, binding to GAG did not alter the binding of m971 (Fig. 5), as would be expected from the crystal structure because the two sites are nonoverlapping.

## Discussion

CD22, a surface membrane glycoprotein expressed on developing and mature human B cells, represents a validated antigen for CAR-T cell immunotherapy against B-ALL (18, 19). Here, we have structurally delineated the epitope of m971 by cocrystallization of a truncated form of CD22 containing its two most membrane-proximal domains and m971 Fab. This structure revealed that m971 binds to the most membrane-proximal Ig-like domain (d7) in CD22. Our biophysical and structural data also indicate that the epitope of m971 does not include the N634-linked glycan. As opposed to m971, the therapeutic antibody epratuzumab showed glycan dependency for binding to CD22 ECD (11). This hindrance dependency of mAbs for binding to glycans on the surface of the glycoprotein is particularly relevant because variable glycoforms on N-glycans, such as truncations or modified branching patterns, have been observed in cancer cells (20, 21). However, it is still unknown how the glycosylation patterns of CD22 vary among B cell-derived malignancies and patients.

**Figure 6:**
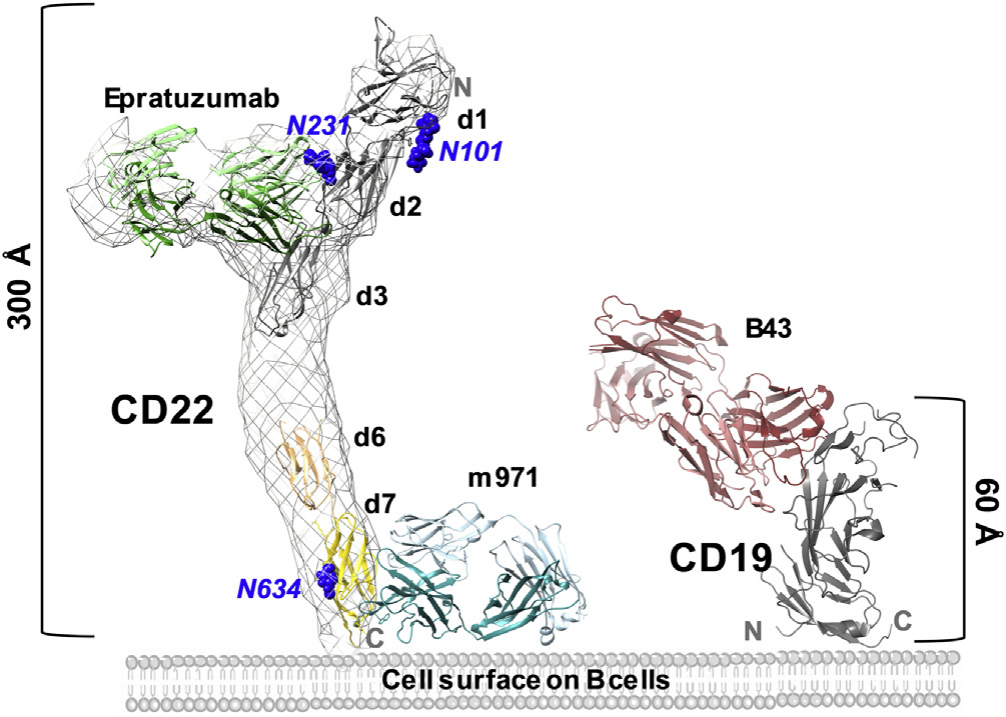
**Structural representation of CD22 and CD19 targets on the B cell surface.** Model depicting the size difference of the extracellular domains of CD22 and CD19. The CD22 ECD map determined by electron microscopy (EMD-8705) (11) is shown as a *gray surface*, fitted with the crystal structure of CD22_d1–d3_ in complex with epratuzumab Fab (PDB ID: 5VL3) (11) and CD22_d6–d7_ in complex with m971 Fab. The crystal structure of the CD19 ECD in complex with B43 Fab (PBD ID: 6AL5) (41) is represented as a *cartoon*. The electron microscopy map and crystal structures were rendered using UCSF Chimera. ECD, extracellular domain; Fab, fragment antigen-binding.

For the efficacy of CAR-T cell immunotherapy, it has been shown that proximity of cell membranes at the immune synapse between the T cell and tumor cell can be an important factor (22, 23, 24). This principle is based on the kinetic segregation model for T-cell receptor signaling (25). For this reason, the size of the target antigen on the tumor cell can play an important role for CAR efficacy (26). In light of these findings, it is interesting to compare the size of the extracellular portion of CD22 and CD19, two major antigen targets on B cells for immunotherapy in B-ALL. While the ECD of CD19 is composed of a hybrid-type Ig-like domain extending around 60 Å, CD22 extends 300 Å with its seven Ig-like domains (d1–d7) (Fig. 6). As previously reported, targeting the membrane-proximal Ig-like domain on CD22 with m971-derived CAR-T cells showed comparable antileukemic activity to anti-CD19 CAR-T cells (10). Moreover, m971-derived CAR-T cell showed higher activity than anti-CD22 CAR-T cells of similar affinity targeting membrane-distal regions (9, 10). A similar epitope location dependency was reported for CAR-T cells against mesothelin (MSLN) (27). Indeed, CARs targeting the membrane-proximal region on MSLN showed increased cytotoxic and cytokine secretion compared with membrane-distal epitope-targeting CARs. As with anti-MSLN CARs, we cannot rule out the possibility that membrane-proximal regions on the targeted CD22 are structurally rigid and thus enable better signal transduction. In addition, the membrane-distal epitopes on CD22 may be participating in interactions with *cis*-ligands and *trans*-ligands, which might impede CAR binding. Although these examples across three antigens highlight the value of targeting membrane-proximal extracellular epitopes for CAR-T efficacy, further work will be required to determine whether these observations translate into a generalizable model that membrane proximal-targeting CARs are consistently better than membrane distal-targeting CARs.

Antigen-binding affinity should also be considered in the design of CAR-T cells (3, 28). Indeed, CAR-T cells possessing low micromolar affinity (1 μM) have been shown to be capable of lysing cells overexpressing target antigens while sparing those with much lower densities (29). On the other hand, early studies using human epidermal growth factor receptor 2-CAR-T cells with varying affinities demonstrated that the activation threshold of CAR-T cells is inversely correlated with the binding affinity (30). By functionally investigating the affinity of various mutants of m971 Fab designed based on our co-crystal structure, we developed a range of binding affinities for m971 variants toward CD22. In this case, the 5-fold gain in affinity for CD22 of Y^H52A^R m971-derived CAR-T cells was not transduced in altered cell killing.

For the generation of more effective mAbs, it is relevant to understand the biological environment in which the target molecule is present. Antibodies should indeed recognize their epitopes in the context of their biological environments. For this reason, binding of mAbs to domains close to the cell surface can be influenced by the presence of phospholipids and glycolipids. Indeed, previously characterized mAbs interacting with membrane-proximal epitopes, such as the anti-HIV-1 envelope glycoprotein 41 broadly neutralizing antibodies 2F5 (31) and 4E10 (32), were found to directly interact with membrane lipid components. Despite the fact that m971 was selected against a recombinant CD22 by phage display (14, 33), the presence of sulfate ions near basic surface patches close to the base of the CD22–m971 complex raise the question of whether positively charged residues on HCDR2 are important for mediating additional interactions with phosphoinositide head groups at the surface of the cell membrane (Fig. 6). Our molecular data suggest that for membrane proximal-targeting CAR-binding domains, a future area of investigation will be to determine whether interaction with lipids on the cell membrane play a role in influencing CAR activity.

## Experimental procedures

### CD22_d6–d7_ and CD22_d1–d7_ construct design, expression, and purification

Full-length CD22 ECD (CD22_d1–d7,_ residues 20–687) was designed, expressed, and purified as previously described (11). The CD22_d6–d7_ (residues 505–688) construct was generated from CD22_d1–d7_ using In-Fusion HD Cloning Kit (Takara) following the manufacturer’s instructions. The construct was cloned into the pHLsec vector (34) using restriction enzymes AgeI and KpnI, such that a 6× His tag was added at the C terminus of the construct to facilitate affinity purification. CD22_d6–d7_ was transiently transfected using the transfection reagent FectoPRO (Polyplus Transfection) in HEK 293 Gnt I^−/−^ (HEK 293S; ATCC CRL-3022) suspension cells for protein expression. HEK 293S cells were incubated at 37 °C, 180 rpm, 8% CO_2_ in a Multitron Pro shaker (Infors HT) for 6 to 7 days. After harvesting the cells by centrifugation, supernatants were retained and filtered using a 0.22 μm Steritop filter (EMD Millipore). Supernatants were passed through a HisTrap Ni-NTA column (GE Healthcare) at 4 ml min^−^^1^. The column was washed with 20 mM Tris, pH 8.0, 150 mM NaCl, and 5 mM imidazole buffer before elution with an increasing gradient of imidazole (up to 500 mM). Fractions containing CD22_d7–d6_ were pooled, concentrated, and separated on a Superdex 200 Increase size exclusion column (GE Healthcare) at 0.5 ml min^−^^1^ in 20 mM Tris, pH 8.0, 150 mM NaCl buffer to achieve size homogeneity.

### m971 Fab construct design, expression, and purification

The m971 IgG construct was kindly provided by Dr Yan Feng. The heavy chain and light chain of m971 Fab were subcloned into the pHLsec vector (34) using In-Fusion HD Cloning Kit (Takara) following the manufacturer’s instructions. Point mutations S^H31B^Q, Y^H52A^R, Y^H52A^E, S^H53^A, or L^H100^Q were introduced by PCR using the QuikChange Site-Directed Mutagenesis protocol. The heavy chain and light chain of the Fab were cotransfected in HEK 293F cells (Thermo Fisher Scientific) using FectoPRO (Polyplus Transfection) at a 2:1 ratio. Cells were transfected at a cell density of 0.8 × 10^6^ cells ml^−^^1^ and incubated at 37 °C, 125 rpm, 8% CO_2_ in a Multitron Pro shaker (Infors HT) for 5 to 7 days. Cells were harvested, and supernatants retained and filtered with a 0.22 μm membrane (EMD Millipore). Supernatants were flowed through a KappaSelect affinity column (GE Healthcare) using an AKTA Start chromatography system (GE Healthcare) and eluted with 100 mM glycine, pH 2.2. Eluted fractions were immediately neutralized with 1 M Tris HCl, pH 9.0. Fractions containing protein were pooled and run through a desalting column to change the sample buffer into 20 mM sodium acetate, pH 5.6. Ion-exchange chromatography was performed using a Mono S column (GE Healthcare) and eluted with a potassium chloride gradient. Fractions were pooled, concentrated, and flowed on a Superdex 200 Increase gel filtration column (GE Healthcare) to obtain purified samples.

### CD22_d6–d7_–Fab complex expression and purification

CD22_d6–d7_ was transiently cotransfected with the heavy chain and light chain of the m971 Fab into HEK 293S cells at a 1:1.5:1 ratio of CD22:HC:LC DNA and using FectoPRO (Polyplus Transfection). Cells were cotransfected at a cell density of 0.8 × 10^6^ cells ml^−^^1^ and incubated at 37 °C, 125 rpm, 8% CO_2_ in a Multitron Pro shaker (Infors HT) for 5 to 7 days. After harvesting the cells by centrifugation at 6371*g* for 20 min, supernatants were retained and filtered using a 0.22 μm Steritop filter (EMD Millipore). Supernatants were passed through a HisTrap Ni-NTA column (GE Healthcare) at 4 ml min^−^^1^. The column was washed with 20 mM Tris, pH 8.0, 150 mM NaCl, and 5 mM imidazole buffer before elution with an increasing gradient of imidazole (up to 500 mM). Fractions containing protein were pooled, concentrated, and separated on a Superdex 200 Increase size exclusion column (GE Healthcare) at 0.5 ml min^−^^1^ in 20 mM Tris, pH 8.0, 150 mM NaCl buffer to achieve size homogeneity. To obtain deglycosylated samples, purified CD22_d6–d7_–m971 Fab complex was treated with the enzyme Endo H (New England Biolabs) for 1 h at 37 °C. The deglycosylated complex was purified further *via* a second Superdex 200 Increase size-exclusion column (GE Healthcare) at 0.5 ml min^−^^1^ in 20 mM Tris, pH 8.0, and 150 mM NaCl buffer.

### CD22 and GAG complex formation

CD22_d1–d7_ and CD22_d6–d7_ were mixed and incubated for 30 min on ice with 10× molar excess of heparin sodium salt GAG of 14 to 16 kDa molecular weight (Toronto Research Chemicals, H245800). Samples were run on a Superdex 200 Increase size exclusion column (GE Healthcare) at 0.5 ml min^−^^1^ in 20 mM Tris, pH 8.0, and 150 mM NaCl buffer. Epratuzumab Fab was used as a control protein.

### Melting and aggregation temperature measurements

The T_m_ and the T_onset_ were simultaneously assessed by monitoring the intrinsic fluorescence emission and SLS (266 nm) using a UNit instrument (Unchained Labs). The T_m_ was determined as the inflection point in the primary data plotted as a function of the temperature, and the T_onset_ was measured by determining the temperature at which the SLS signal reaches a threshold that is 10% of its maximum value. Thermal stability was analyzed using a temperature ramp from 20 to 85 °C using 1 °C increments, with an equilibration time of 60 s before each measurement. Samples were prepared at 1 mg ml^−^^1^. Measurements were made in 100 mM Tris (pH 9.0), Hepes (pH 7.4), and sodium acetate (pH 5.6) buffers with 150 mM NaCl. Measurements were made in duplicates and averaged, and standard errors were calculated using GraphPad Prism v6.

### BLI

The binding affinities of WT m971 Fab or m971 Fab mutants to different CD22 ECD (CD22_F_, CD22_S_ and CD22_N634A_) were measured by BLI using the Octet RED96 BLI system (Pall ForteBio). Ni-NTA biosensors were hydrated in 1× kinetics buffer (20 mM Tris, pH 7.4, 0.002% Tween, 0.01% BSA) and loaded with 25 ng μl^−^^1^ of CD22_d1–d7_ for 60 s at 1000 rpm. Biosensors were then transferred into wells containing 1× kinetics buffer to baseline for 60 s before being transferred into wells containing a serial dilution of Fab starting at 200 nM and decreasing to 25 nM. The 180 s association phase was subsequently followed by a 180 s dissociation step in 1× kinetics. Analysis was performed using the Octet software, with a 1:1 fit model. All experiments were repeated in duplicates, values were averaged, and standard errors were calculated.

### m971 CAR-T cell production and cellular cytotoxicity assay

m971 CAR-T cells were produced by lentiviral transduction of human Jurkat T cell lines. *Lentivirus* was packaged in HEK 293T cells by cotransfecting pLVX-IRES-m971 CAR, psPAX2, and pMD2.G plasmids. Virus was purified and concentrated by the Retro-X Concentrator (Takara, Clontech). Human Jurkat T cell lines were transduced by the spinoculation method. Briefly, the virus was attached into RetroNectin (Takara, Clontech) coated non-tissue culture-treated 6-well plates by centrifugation at 2000*g*, 30 °C for 2 h. Then, the supernatant was aspired and Jurkat T cells were added into plates by centrifugation at 1000*g* at 30 °C for 10 min. To improve CAR expression, the next day, Jurkat T cells were subjected to the second spinoculation. For cell killing, CAR-Jurkat T cells were incubated with Raji cells (10^4^ cells) in triplicates with a E:T ratio of 10:1 for 24 h. Killing of Raji cells was detected by the Promega CytoTox-Glo Cytotoxicity Assay (Cat. No. G9290) with the following formula: lysis% = 100 × (RLU_E+T_-RLU_E_-RLU_T_)/(RLU_max_-RLU_T_), wherein RLU_E+T_ refers to the luminescence intensity of wells with effector cell incubation with target cells and RLU_E_ and RLU_T_ are for spontaneous signals for effector cells and target cells alone.

### Crystallization and X-ray diffraction

To obtain crystals of m971, the purified Fab was mixed with 2 to 5× molar excess of anti-kappa V_H_H domain (catalog number: 1033270500, BAC BV (Thermo Fisher Scientific)) as described previously (15) and run on a gel filtration chromatography. The purified m971 Fab-V_H_H complex was concentrated to 9 mg ml^−^^1^ in 20 mM sodium acetate, pH 5.6. Crystals of the m971 Fab-V_H_H complex were obtained by sitting-drop vapor diffusion at 20 °C in 0.2 M lithium acetate, and 20% (w/v) PEG3350 in 96-well plates after mixing 0.15 μl of protein and 0.15 μl of solution using an Oryx4 crystallization robot (Douglas Instruments). Crystals were cryoprotected by soaking them in the mother liquor solution containing 20% glycerol and flash-cooled in liquid nitrogen. X-ray diffraction data were collected at the 08ID synchrotron beamline at the Canadian Light Source. The dataset of the m971 Fab-V_H_H complex was processed in space group P3_1_ using XDS (35). The structure was solved by molecular replacement in Phaser using the variable and constant domains of Fab fragments from our internal database as search models and refined by manual building in Coot (36) and using phenix.refine (37).

The purified CD22_d6–d7_–m971 Fab complex was concentrated to 12 mg ml^−^^1^ in 20 mM Tris, pH 8.0, and 150 mM NaCl. Crystals of the CD22_d6–d7_–m971 Fab complex were obtained by sitting-drop vapor diffusion at 20 °C in 2 M ammonium sulfate and 0.1 M Hepes, pH 7.5, in 96-well plates after mixing 0.15 μl of protein and 0.15 μl of solution using an Oryx4 crystallization robot (Douglas Instruments). Crystals were cryoprotected by soaking them in the mother liquor solution containing 20% glycerol and flash-cooled in liquid nitrogen. X-ray diffraction data were collected at the 08ID synchrotron beamline at the Canadian Light Source. The dataset of the CD22_d6–d7_–m971 Fab complex was processed in space group I222 using XDS (35). The structure was solved by molecular replacement in Phaser using the structure of unliganded m971 Fab as a starting model and refined by manual building in Coot (36) and using phenix.refine (37). Data collection and refinement statistics are reported in Table 1. All software programs were accessed through SBGrid (38).

## Data availability

The crystal structures have been deposited in the Protein Data Bank, www.rcsb.org (PDB ID: 7O4Y and 7O52).

## Supporting information

This article contains supporting information (39, 40, 42).

## Conflict of interest

The authors declare that they have no conflicts of interest with the contents of this article.
