# Supplementary material for: Structural details of monoclonal antibody m971 recognition of the membrane-proximal domain of CD22
Source: J Biol Chem. 2021 Jul 14;297(2):100966. doi: 10.1016/j.jbc.2021.100966 (PMC8353475; doi:10.1016/j.jbc.2021.100966)
Supplement: Supplemental Figures S1–S6 and Table S1 [file mmc1.pdf]

Supporting information

Supporting Figure 1

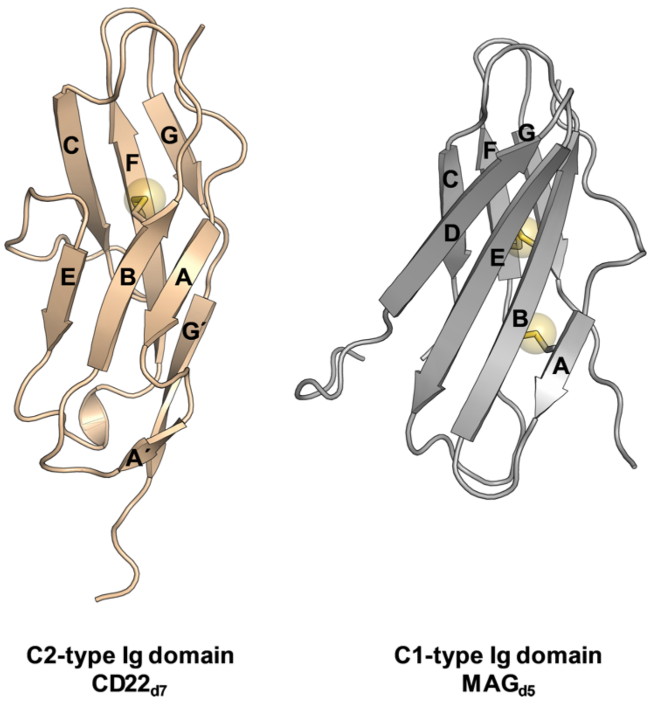

Supporting Figure 1. Comparison of the three-dimensional structures of CD22 and MAG proximal Ig domains. Crystal structures of d7 and d5 proximal Ig-like domains of CD22 and MAG, respectively. C1- and C2-type Ig folding contain different β-strand topology of strands D and C´.

Supporting Figure 2

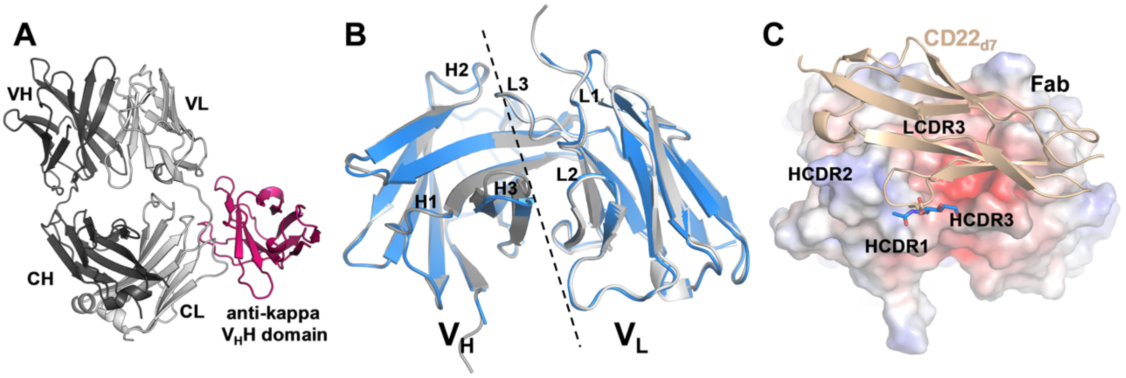

Supporting Figure 2. Variable region of m971 Fab. A) Crystal structure of m971 Fab (grey) in complex with the anti-kappa V_HH domain (magenta). The anti-kappa V_HH domain interacts with the Fab constant region of the light chain (CL). B) Superposition of the crystal structures of the variable domain of m971 Fab unliganded (in blue) and CD22-bound (grey). Except for the HCDR3 loop, no significant changes are observed in the CDRs, indicating that m971 is largely in a predisposed conformation to bind CD22 d7. C) Electrostatic surface representation of the m971 paratope. The d7 domain of CD22 is shown as wheat secondary structure. The calculation of the surface electrostatics was made with the APBS software (1) and prepared using Pymol (2) and displayed on a scale of -5 kT/e (red) to 5 kT/e (blue).

Supporting Figure 3

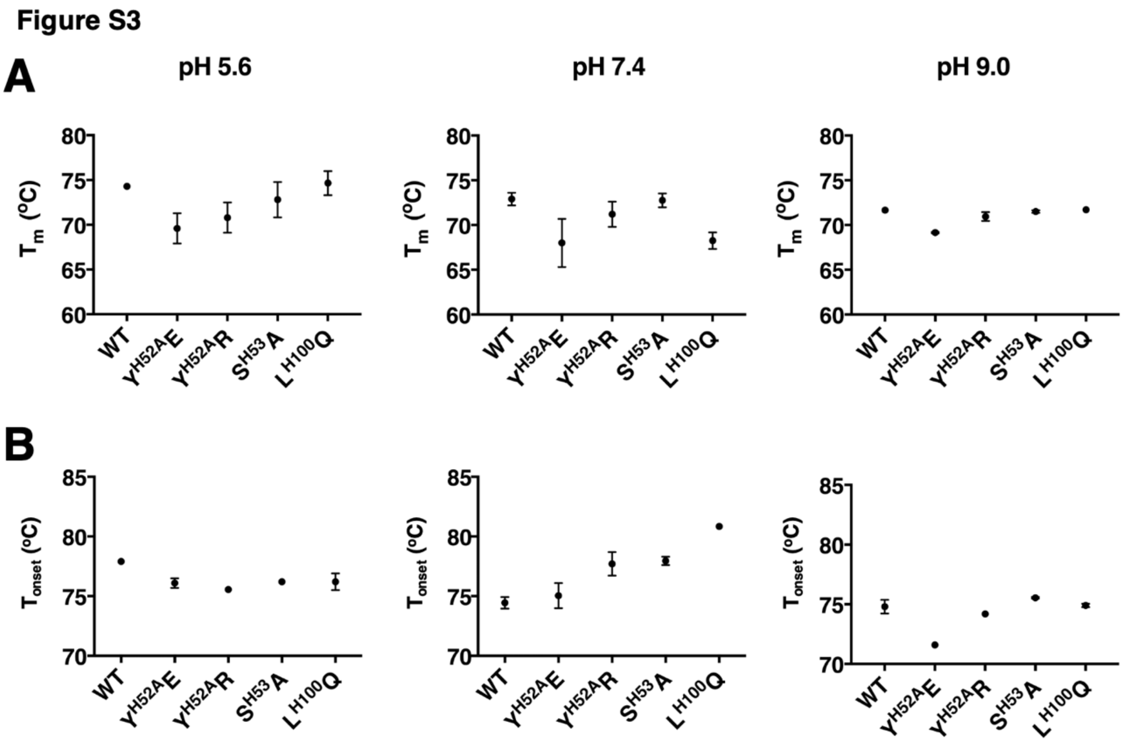

Supporting Figure 3. Thermal stability of WT and mutant m971 Fabs at different pH's. A) Melting temperature (T_m) calculated from intrinsic fluorescence. B) Onset aggregation temperature (T_{onset}) calculated from static light scattering.

Supporting Figure 4

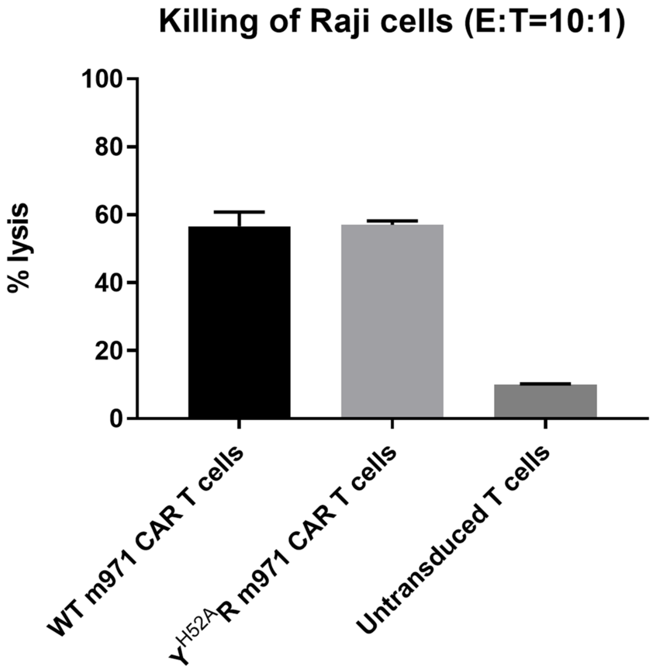

Supporting Figure 4. Lysis of Raji cells by wild type (wt) and Y^{H52A}R mutant m971 CAR-T cells. WT m971 and Y^{H52A}R m971 scFv were incorporated into the 2nd generation CAR with CD28 transmembrane domain and intracellular 4-1BB and CD3 ζ domain, which were expressed on Jurkat T cells surface by lentiviral-mediated transduction. CAR-T cell killing of Raji cells was detected by the Promega CytoTox-Glo™ Cytotoxicity Assay kit. Untransduced T cells were used as a negative control.

Supporting Figure 5

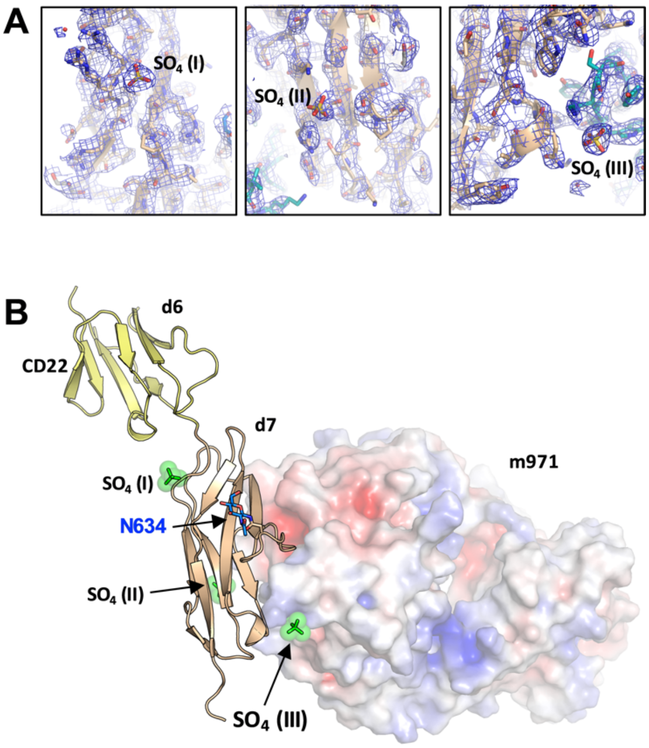

Supporting Figure 5. Sulphate ions in the CD22$_{d6\text{-}d7}$-m971 Fab co-crystal structure. A) Representative electron density showing the sulphate ion sites I (left), II (middle) and III (right) on the surface of CD22 (in wheat color). The electron density was calculated from a composite omit map and contoured at 1σ. B) Crystal structure of CD22$_{d6\text{-}d7}$ in complex with m971 Fab. Electrostatic surface of the m971 Fab is represented. SO_4 III is located at the interface between CD22 and m971. The calculation of the surface electrostatics was made with the APBS software (1) and prepared using Pymol (2) and displayed on a scale of -5 kT/e (red) to 5 kT/e (blue).

Supporting Figure 6

```
hCD22   1   MHLLGPWLLLLVLEY--LAFSDSSKWVFEHPETLYAWEGACVWIPCTYRAL--DGDLESF     56
mCD22   1   MRVHYLWLLLILGHAASAQYSSANDWTVDHPQTLFAWEGACIRIPCKYKTPLPKARLDNI     60
rCD22   1   MCVPCPWLLLILGHVAS--FRSATGWSVEHPQTLFAWEGACIQIPCKYKVPKPRSRLDSI     58
            * :   ****:: .      : .:. * .:**:**:******: ***.*:.    . *:.:

hCD22  57   ILFHNPEYNKNTSKFDGTRLYESTKD----GKVPSEQKRVQFLGDKNKNCTLSIHPVHLN    112
mCD22  61   LLFQNYEFDKATKKFKGTVLYNKA----EPELYPPKQRRVTFLGNSIDNCTLKIHPIRAN    116
rCD22  59   LLFQNYTFDNTTRDFTGKVLYNNTKASIETELYFSQQDRVTFLGNRSNNCTLKIDPIHAN    118
            :**:*   ::: * .* *. **:.:               :* ** ***:  .****.*.*:: *

hCD22 113   DSGQLGLRMESKTEKWMERIHLNVSERPFFPPHIQLPPEIQESQEVTLTCLLNFSCYGYPI   172
mCD22 117   DSGNLGLRMTAGTERWMEPIHLNVSEKPFQPYIQMPSEIRESQSVTLTCGLNFSCFEYDI   176
rCD22 119   DSGKLGLRLISGTDKWMEHIHLNVSERPFQPYIQLPSEIRESQRVSLTCGLNFTCFGYDI   178
            ***:****: : *::*** *******:** *:**:* **:*** *:*** ***:*: * *

hCD22 173   QLQWLLEGVPMRQAAV------TSTSLTIKSVFTRSELKFSPQWSHHGKIVTCQLQDADG   226
mCD22 177   LLQWFLEDSKITSVTPSVTSITSSVTSSIKNVYTESKLTFQPKWTDHGKSVKCQVQHSS-   235
rCD22 179   SLRWSLDGSEVSPV-------TSSITSSVENVYTESKLTFQPKWTDHGKSMMCQVWHFS-   230
             *:* *:.  :  .         :* : :::.*:*.*:*.*.*:*:.*** : **: . .

hCD22 227   KFLSNDTVQLNVKHTPKLEIKVTPSDAIVREGDSVTMTCEVSSSNPEYT--TVSWLKDGT   284
mCD22 236   EVLSERTVRLDVKYTPKLEIKVNPTE--VEKNNSVTMTCRVNSSNPKLRTVAVSWFKDGR   293
rCD22 231   QVLSERTVRLDVKYTPKLQITVNPTE--VKEGNSVTMTCQVNSSNPSLSTKKVSWFKDGS   288
            :.**: **:*:**:****:*.*.*::  *.:.:******.*.****.      ***:***

hCD22 285   SLKKQN------TFTLNLREVTKDQSGKYCCQVSNDVGPGRSEEVFLQVQYAPEPSTVQI   338
mCD22 294   PLEDQELEQEQQMSKLILHSVTKDMRGKYRCQASNDIGPGESEEVELTVHYAPEPSRVHI   353
rCD22 289   FLKEQQ-----EQEELTLHSVTKDMRGKYQCRASNNIGQGQSKEVALTVLYPPEPSKVHI   343
             *:.*:            * *:.****  *** *:.**::* *.*:** * * * **** *:*

hCD22 339   LHSPAVEGSQVEFLCMSLANPLPTNYTWYHNGKEMQGRTEEKVHIPKILPWHAGTYSCVA   398
mCD22 354   YPSPAEEGQSVELICESLASPSATNYTWYHNRKPIPGDTQEKLRIPKVSPWHAGNYSCLA   413
rCD22 344   YPSPAEEGQSVELICDSLASPRATNYTWYHNGEMVPGASHEKLQISNVSLWHAGKYSCLA   403
               *** **..**::* ***.*  ******** : : * :.**::* ::  ****.***:*

hCD22 399   ENILGTGQRGPGAELDVQYPPKKVTTVIQNPMPIREGDTVTLSCNYNSSNPSVTRYEWKP   458
mCD22 414   ENRLGHGKIDQEAKLDVHYAPKAVTTVIQSFTPILEGDSVTLVCRYNSSNPDVTSYRWNP   473
rCD22 404   ENRLGCGKIEQEAELDVHYVPKAVTTVIQSVTPIREGDSVTLDCRYNSSNPEVTKYEWSP   463
            ** ** *:      *:***:* ** ******.  ** ***:*** *.******.** *.*.*

hCD22 459   HGAWEEPSLGVLKIQNVGWDNTTIACAACNSWCSWASPVALNVQYAPRDVRVRKIKPLSE   518
mCD22 474   QGSGSVLKPGVLRIQKVTWDSMPVSCAACNHKCSWALPVILNVHYAPRDVKVLKVSPASE   533
rCD22 464   QGSGSEVTPGVLRIQKATWDSPPIKCAACNYKCSWSFPVSLNVHYAPRAVKILKVSPASE   523
            :*:  .    . ***:**:. **.   : *****   ***: ** ***:**** *:: *:.* **

hCD22 519   IHSGNSVSLQCDFSSSHPKEVQFFWEKNGRLLGKESQLNFDSISPEDAGSYSCWVNNSIG   578
mCD22 534   IRAGQRVLLQCDFAESNPAEVRFFWKKNGSLVQEGRYLSFGSVSPEDSGNYNCMVNNSIG   593
rCD22 524   IHAGQHVLLQCDFSGSFPTEVRFFWKKNGNLVQEGRYLNFSSISPEDSGNYNCMVRNSIG   583
            *:*:  * *****: * * **:***:*** *: :   *.*.*:****:*.*.* *.****

hCD22 579   QTASKAWTLEVLYAPRRLRVSMSPGDQVMEGKSATLTCESDANPPVSHYTWFDWNNQSLP   638
mCD22 594   ETLSQAWNLQVLYAPRRLRVSISPGDHVMEGKKATLSCESDANPPISQYTWFDSSGQDLH   653
rCD22 584   ETSSQAWSLQVLYAPRRLRVSISPGDSVMEGKKATLSCESDAHPPVFKYIWFDANDQDLH   643
            :* *:**.*:*************:**** *****.***:*****:**: :* ***  :* *** ..*.*

hCD22 639   YHSQKLRLEPVKVQHSGAYWCQGTNSVGKGRSPLSTLTVYYSPETIGRRVAVGLGSCLAI   698
mCD22 654   SSGQKLRLEPLEVQHTGSYRCKGTNGIGTGESPPSTLTVYYSPETIGKRVALGLGFCLTI   713
rCD22 644   FSGQKLRLEPLRVQHTGSYRCQGINELGTGKSPPSTLTVYYSPETIGKRVALGLGFCLAI   703
              .*******:.***:*:* *:* *  :*.*.** *************:***:*** **:*

hCD22 699   LILAICGLKLQRRWKRTQSQQGLQENSSGQSFFVRNKKVRRAPLSEGPHSLGCYNPMMED   758
mCD22 714   CILAIWGMKIQKKWKQNRSQQGLQENSSGQSFFVRNKKARRTPLSEGPQSQGCYNPAMDD   773
rCD22 704   CILAIWGMKIQKKWKHNRSQQGPQDNSSGQSFFVRNKKARRTRLTEGPQSQGCYNLAMDD   763
             **** *:*:*::**:.:****:************* .**: *:***:* ****  *:*

hCD22 759   GISYTTLRFPEMNIPRTGDAESSEMQRPPPDCDDTVTYSALHKRQVGDYENVIPDFPEDE   818
mCD22 774   TVSYAILRFPESDMHNAGDAGTPATQAPPPNNSDSVTYSVIQKRPMGDYENVNPSCPEDE   833
rCD22 764   TVSYAVLRFPESDTHGAGGARSPATQGPPPNDDDTVTYSVLQKRNMGDYENVSPNCPEDE   823
             :**: *****  :   :*.* :    * ***: .*:****.::** :****** *.  ****

hCD22 819   GIHYSELIQFGVGERPQAQENVDYVILKH                                  847
mCD22 834   SIHYSELVQFGAGKRPQAKEDVDYVTLKH                                  862
rCD22 824   SIHYSELVQFGAGKRPQAKEDVDYVTLKH                                  852
            .******:***.*:****:*:**** ***
```

Supporting Figure 6. Sequence alignment of CD22. Amino acid sequence alignment of CD22 from human (hCD22), mouse (mCD22) and rat (rCD22). The red box indicates the putative heparan sulfate binding site, with XBBXBX motif (X, aliphatic residues and B, amino acids R, K or H). Sequence alignment was done using the Clustal Omega software (3).

Supporting Table 1. Residues involved in the m971-CD22 interaction. S= salt bridge; H= hydrogen bond.

Contact type	CD22 residue	BSA (Å^2)
	P623	12
	V624	10
	S625	56
S	H626	71
	Y627	8
H	T628	18
H	D631	16
	W632	35
H	N633	11
H	Q635	62
	S636	28
	L637	32
H	P638	109
H	Y639	70
HS	H640	136
H	S641	52
	Q642	3
	R645	11
	L646	22
HS	E647	65
	P648	43
	K650	15
	Q652	36
H	H653	57
H	Y657	12

	**Total**	**990**
	**m971 HC residue**	**BSA (Å^2)**
H	N32	32
	A35	1
H	R50	32
H	Y52	38
	R52b	78
H	S53	64
	K54	65
H	W55	25
H	Y56	99
	N57	37
H	D58	33
	Y59	28
HS	K64	30
	T97	20
	G98	12
HS	D99	74
	L100	26
HS	D100b	28
	**Total**	**722**
	**m971 LC residue**	**BSA (Å^2)**
	W30	71
H	Y32	60
	S91	11
H	Y92	78
	S93	15

	I94	41
	**Total**	**276**

References

1. Unni, S., Huang, Y., Hanson, R. M., Tobias, M., Krishnan, S., Li, W. W., Nielsen, J. E., and Baker, N. A. (2011) Web servers and services for electrostatics calculations with APBS and PDB2PQR. *J. Comput. Chem.* 10.1002/jcc.21720
2. Schrödinger, L. (2015) The PyMol Molecular Graphics System, Versión 1.8. *Thomas Hold.* 10.1007/s13398-014-0173-7.2
3. Sievers, F., and Higgins, D. G. (2014) Clustal omega, accurate alignment of very large numbers of sequences. *Methods Mol. Biol.* 10.1007/978-1-62703-646-7_6
